# Supplementary material for: Heat adaptation of phage T7 under an extended genetic code
Source: Virus Evol. 2021 Dec 1;7(2):veab100. doi: 10.1093/ve/veab100 (PMC8923235; doi:10.1093/ve/veab100)
Supplement: veab100_Supp [file veab100_supp.zip › Sequencing_Analysis.zip!/Sequencing_Analysis/S1/evidence/JC_53.html]

BRESEQ :: Evidence


| New junction evidence | | | | | | | | | | | |
| --- | --- | --- | --- | --- | --- | --- | --- | --- | --- | --- | --- |
|  | | seq id | position | reads (cov) | reads (cov) | score | skew | freq | annotation | gene | product |
| \* | ? | NC\_001604.1 | = 9895 | 4719 (1.120) | 64 (0.020) | 49/592 | 27.8 | 1.3% | intergenic (–/–) | *–/–* | –/– |
|  | ? | NC\_001604.1 | 10215 = | 4867 (1.180) |  |  |  |  | intergenic (–/–) | *–/–* | –/– |
| Rejected: Coverage evenness skew score above cutoff. | | | | | | | | | | | |
| Rejected: Frequency below/above cutoff threshold. | | | | | | | | | | | |

Only 100 of 234 total aligned reads displayed.  

`GGCTCTAAGCTGAAAGTTAAATATTCTCTGGTTCCATACAAGTGGAACACTGCTGTAGGTGCGAGCGTTAAGCTGCAACTGGAATCCGTGATGCTGGTCGAACTGGCTACCTTTGGTGGCGGTGAAGACGATTGGGCTGACGAAGTTGAAGAGAACGGCTATGTTGCCTCTGGTTCTGCCAAAGCGAGCAAACCACGCGACGAAGAAAGCTGGGACGAAGACGACGAAGAGTCCGAGGAAGCAGACGAAGACGGAGACTTCTAAGTGGAACTGCGGGAGAAAATCCTTGAGCGAATCAAGG‑T‑G‑‑‑‑‑‑‑‑‑‑‑‑‑‑‑‑‑‑‑‑‑‑‑‑‑‑‑‑‑‑‑‑‑‑‑‑‑‑‑‑‑‑‑‑‑‑‑‑‑‑‑‑‑‑‑‑‑‑‑‑‑‑‑‑‑‑‑‑‑‑‑‑‑‑‑‑‑‑‑‑‑‑‑‑‑‑‑‑‑‑‑‑‑‑‑‑‑‑‑‑‑‑‑‑‑‑‑‑‑‑‑‑‑‑‑‑‑‑‑‑‑‑‑‑‑‑‑‑‑‑‑‑‑‑‑‑‑‑‑‑‑‑‑‑‑‑‑‑‑‑‑‑‑‑‑‑‑‑‑‑‑‑‑‑‑‑‑‑‑‑‑‑‑‑‑‑‑‑‑‑‑‑‑‑‑‑‑‑‑‑‑‑‑‑‑‑‑‑‑‑‑‑‑‑‑‑‑‑‑‑‑‑‑‑‑‑‑‑‑‑‑‑‑‑‑‑‑‑‑‑‑‑‑‑‑‑‑‑‑‑‑‑‑‑‑‑‑‑‑‑‑‑‑‑‑‑‑‑‑‑‑‑‑‑‑‑‑‑‑‑‑‑‑‑‑‑‑‑‑‑‑‑‑‑‑‑‑‑‑‑‑‑‑‑‑‑‑‑‑‑‑‑  >  NC_001604.1/9593‑9895`  
`‑‑‑‑‑‑‑‑‑‑‑‑‑‑‑‑‑‑‑‑‑‑‑‑‑‑‑‑‑‑‑‑‑‑‑‑‑‑‑‑‑‑‑‑‑‑‑‑‑‑‑‑‑‑‑‑‑‑‑‑‑‑‑‑‑‑‑‑‑‑‑‑‑‑‑‑‑‑‑‑‑‑‑‑‑‑‑‑‑‑‑‑‑‑‑‑‑‑‑‑‑‑‑‑‑‑‑‑‑‑‑‑‑‑‑‑‑‑‑‑‑‑‑‑‑‑‑‑‑‑‑‑‑‑‑‑‑‑‑‑‑‑‑‑‑‑‑‑‑‑‑‑‑‑‑‑‑‑‑‑‑‑‑‑‑‑‑‑‑‑‑‑‑‑‑‑‑‑‑‑‑‑‑‑‑‑‑‑‑‑‑‑‑‑‑‑‑‑‑‑‑‑‑‑‑‑‑‑‑‑‑‑‑‑‑‑‑‑‑‑‑‑‑‑‑‑‑‑‑‑‑‑‑‑‑‑‑‑‑‑‑‑‑‑‑‑‑‑‑‑‑‑‑‑‑‑‑‑‑‑‑‑‑‑‑‑‑‑‑‑‑‑‑‑‑‑‑‑‑‑‑‑‑‑‑‑‑‑‑‑‑‑‑‑‑‑‑‑‑gg‑t‑gTCTCCCAACAGACTATTTGTGATATACGCAAAGGGAGGCGACATGGCAGGTTACGGCGCTAAAGGAATCCGAAAGGTTGGAGCGTTTCGCTCTGGCC‑‑TAGAGGACAAGGTTTCAAAGCAGTTGGAATCAAAAGGTATTAAATTCGAGTATGAAGAGTGGAAAGTGCCTTATGTAATTCCGGCGAGCAATCACACTTACACTCCAGACTTCTTACTTCCAAACGGTATATTCGTTGAGACAAAGGGTCTGTGGGAAAGCGATGATAGAAAGAAGCACTTATTAATTAGGGAGCAGCACCCC  >  NC_001604.1/10215‑10514`  

                                                                                                                                                                                                                                                                                                                                                                                                                                                                                                                                                                                                                                 
`gtgtataagagacagGTTAAATATTCTCTGGTTCCATACAAGTGGAACACTGCTGTAGGTGCGAGCGTTAAGCTGAAACTGGAATCCGTGATGCTGGTCGAACTGGCTACCTTTGGTGGCGGTGAAGACGATTGGGCTGACGAAGTTGAAGAGAACGGCTATGTTGCCTCTGGTTCTGCCAAAGCGAGCAAACCACGCGACGAAGAAAGCTGGGACGAAGACGACGAAGAGTCCGAGGAAGCAGACGAAGACGGAGACTTCTAAGTGGAACTGCGGGAGAAAATCCTTGAGCGAATCAAGG                                                                                                                                                                                                                                                                                                                    <  1:439668/286‑1`  
`gtcttTAAGCTGAAAGTGAAATATTCTCTGGTTCCATACAAGTAGAACACTGCTGTAGGTGCGAGCGTTAAGCTGCAACTGGAATCCGTGATGCTGGTCGAACTGGCTACCTTTTGTGGCGGTGAAGACGATTGGGCTGACGAAGTTGAAGAGAACGGCTATGTTGCCTCTGGTTCTGCCAAAGCGAGCAAACCACGCGACGAAGAAAGCTGGGACGAAGACGACGAAGAGTCCGAGGAAGCAGACGAAGACGGAGACTTCTAAGTGGAACTGCGGGAGAAAATCCTTGAGCGAATCAAGG                                                                                                                                                                                                                                                                                                                    <  1:410114/296‑1`  
`ggcgtTAAGCTGAAAGTTAAATATTCTCTGGTTCCATACAAGTGGAACACTGCTGTAGGTGCGAGCGTTAAGCTGCAACTGGAATCCGTGATGCTGGTCGAACTGGCTACCTTTGGTGGCGGTGAAGACGATTGGGCTGACGAAGTTGAAGAGAACGGCTATGTTGCCTCTGGTTCTGCCAAAGCGAGCAAACCACGCGACGAAGAAAGCTGGGACGAAGACGACGAAGAGTCCGAGGAAGCAGACGAAGACGGAGACTTCTAAGTGGAACTGCGGGAGAAAATCCTTGAGCGAATCAAGG                                                                                                                                                                                                                                                                                                                    <  1:512128/296‑1`  
`gcccggataggtgtagaagagacagCTCTGGGTCCATACAAGTGGAAAACTGCTGTAGGTGCGAGCGGTAAGCTGCAACTGGAATCCGTGATGCTGGTCGAACTGGCTACCTTTGGTGGCGGTGAAGACGATTGGGCTGACGAAGTTGAAGAGAACGGCTATGTTGCCTCTGGTTCTGCCAAAGCGAGCAAACCACGCGACGAAGAAAGCTGGGACGAAGACGACGAAGAGTCCGAGGAAGCAGACGAAGACGGAGACTTCTAAGTGGAACTGCGGGAGAAAATCCTTGAGCGAATCAAGG                                                                                                                                                                                                                                                                                                                    <  1:357849/276‑1`  
`GGCTCTAAGCTGAAAGTTAAATATTTTCTGGTTCCATACAAGTGGAACACTGCTGTAGGTGCGAGCGTTAAGCTGCAACTGGAATCCGTGATGCTGGTCGAACTGGCTACCTTTGGTGGCGGTGAAGACGATTGGGCTGACGAAGTTGAAGAGAACGGCTATGTTGCCTCTGGTTCTGCCAAAGCGAGCAAACCACGCGACGAAGAAAGCTGGGACGAAGACGACGAAGAGTCCGAGGAAGCAGACGAAGACGGAGACTTCTAAGTGGAACTGCGGGAGAAAATCCTTGAGCGAATCAAGG                                                                                                                                                                                                                                                                                                                    <  1:10991/301‑1`  
`GGCTCTAAGCTGAAAGTTAAATATTCTCTGGTTCCATACAAGTGGAACACTGCTGTAGGTGCGAGCGTTAAGCTGCAACTGGAATCCGTGATGCTGGTCGAACTGGCTACCTTTGGTGGCGGTGAAGACGATTGGGCTGACGAAGTTGAAGAGAACGTCTATGTTGCCTCTGGTTCTGCCAAAGCGAGCAAACCACGCGACGAAGAAAGCTGGGACGAAGACGACGAAGAGTCCGAGGAAGCAGACGAAGACGGAGACTTCTAAGTGGAACTGCGGGAGAAAATCCTTGAGCGAATCAAGG                                                                                                                                                                                                                                                                                                                    <  1:170409/301‑1`  
`GGCTCTAAGCTGAAAGTTAAATATTCTCTGGTTCCATACAAGTGGAACACTGCTGTAGGTGCGAGCGTTAAGCTGCAACTGGAATCCGTGATGCTGGTCGAACTGGCTACCTTTGGTGGCGGTGAAGACGATTGGGCTGACGAAGTTGAAGAGAACGGCTATGTTGCCTCTGGTTCTGCCAAAGCGAGCAAACCACGCGACGAAGAAAGCTGGGACGAAGACGACGAAGAGTCCGAGGAAGCAGACGAAGACGGAGACTTCTAAGTGGAACTGCGGGAGAAAATCCTTGAGCGAATCAAGG                                                                                                                                                                                                                                                                                                                    <  1:402835/301‑1`  
`GGCTCTAAGCTGAAAGTTAAATATTCTCTGGTTCCATACAAGTGGAACACTGCTGTAGGTGCGAGCGTTAAGCTGAAACAGGAATCCGTGATGATGGTCGAACTGGCTACCTTTGGTGGCGGTGAAGACGATTGGGCTGACGAAGTTGAAGAGAACGGCTATGTTGCCTCTGGTTCTGCCAAAGCGAGCAAACCACGCGACGAAGAAAGCTGGGACGAAGACGACGAAGAGTCCGAGGAAGCAGACGAAGACGGAGACTTCTAAGTGGAACTGCGGGAGAAAATCCTTGAGCGAATCAAGG                                                                                                                                                                                                                                                                                                                    <  1:499152/301‑1`  
 `gcctTAAGCTGAAAGTTAATTATTCCCTGGTTCCATTCAATTGGTATGCTGCTGTAGGTGCTATCGTTAAGCTGCAACTGGAATCCGTGATGCTGGTCGAACTGGCTACCTTTGGTGGCGGTGAAGACGATTGGGCTGACGAAGTTGAAGAGAACGGCTATGTTGCCTCTGGTTCTGCCAAAGCGAGCAAACCACGCGACGAAGAAAGCTGGGACGAAGACGACGAAGAGTCCGAGGAAGCAGACGAAGACGGAGACTTCTAAGTGGAACTGCGGGAGAAAATCCTTGAGCGAATCAAGG‑T                                                                                                                                                                                                                                                                                                                  <  1:207159/297‑1`  
 `cCTCTAAGCTGAAATTTAAAATTTCTCTGGTTCCATACAAGTTGTACACTTCTGTAGGTGCGAGCGTTAAGCTGCAACTTGATTCTGTGTTTCTGGTCGAACTGGCTACCTTTGGTGGCGGTGAAGACGATTGGGCTGACTAAGTTGAAGAGAACGGCTATGTTGCCTCTGGTTCTGCCAAAGCTAGCAAACCACGCGACGAAGAAAGCTGGGACGAAGACGACGAAGAGTCCGAGGAAGCAGACGAAGACGGAGACTTCTAAGTGGAACTGCGGGAGAAAATCCTTGAGCGAATCAAGG‑T                                                                                                                                                                                                                                                                                                                  <  1:51069/300‑1`  
 `GCTTTAAGCTGAAAGGTAAATATTCGCTGGTTCAATACAAATGGAACACTGCTGTAGGGGCGAGAGTTAAGCTGCAACTGGAATCAGGGATGCTGGTCGAACTGGGTACCTTTGGTGGCGGTTAAGCAGATTGGGCTGACGAAGTTGAAGAGAACGGCTATGTTGCCTCTGGTTCTGCAAAAGCGAGCAAACCACGCGACGAAGAAAGCTGGGACGAAGACGACGTAGAGTCCGAGGAAGCAGACGAAGACGGAGACTTCTAAGTGGAACTGCGGGAGAAAATCCTTGAGCGAATCAAGG‑T                                                                                                                                                                                                                                                                                                                  <  1:316306/301‑1`  
 `GCTCTCAGCTGAAAGTTAAATATTCTCTGGTTCCATACAAGTGGAACACTGCTGTAGGTGCGAGCGTTAAGCTGCAACTGGAATCCGTGATGCTGGTCGAACTGGCTACCTTTGGTGGCGGTGAAGACGATTGGGCTGACGAAGTTGAAGAGAACGGCTATGTTGCCTCTGGTTCTGCCAAAGCGAGCAAACCACGCGACGAAGAAAGCTGGGACGAAGACGACGAAGAGTCCGAGGAAGCAGACGAAGACGGAGACTTCTAAGTGGAATGGCGGGAGAAAATCCTTGAGCGAATCAAGtt                                                                                                                                                                                                                                                                                                                   <  1:464072/301‑3`  
 `GCTCTAAGCTGAAAGTTAAATATTCTCTTGTTCCATACAAGTAGAACACTGCTGTAGGTGCGAGCGTTAAGCTGCAACTGGAATCCGTGATGCTGGTCGAACTGGCTACCTTTGGTGGCGGTGAAGACGATTGGGCTGACGAAGTTGAAGAGAACGGCTATGTTGCCTCTGGTTCGGCCAAAGCGAGCAAACCACGCGACGAAGAAAGCTGGGACGAAGACGACGAAGAGTCCGAGGAAGCAGACGAAGACGGAGACTTCTAAGTGGAACTGCGGGAGAAAATCCTTGAGCGAATCAAGG‑T                                                                                                                                                                                                                                                                                                                  <  1:379458/301‑1`  
 `GCTCTAAGCTGAAAGTTAAATATTCTCTGGTTCCATACAAGTGGAACACTGCTGTAGGTGCGATCGTTAAGCTGCAACTGGAATCCGTGATGCTGGTCGAACTGGCTACCTTTGGTGTCGGTGAAGACGATTGGGCTGACGAAGTTGAAGAGAACGGCTATGTTTCCTCTGGTTCTGCCAAAGCGAGCAAACCACGCGACGAAGAAAGCTGGGACGAAGACGACGAAGAGTCCGAGGAAGCAGACGAAGACGGAGACTTCTAAGTGGAACTGCGGGAGAAAATCCTTGAGCGAATCAAGGg                                                                                                                                                                                                                                                                                                                   >  1:466763/1‑300`  
 `GCTCTAAGCTGAAAGTTAAATATTCTCTGGTTCCATACAAGTGGAACACTGCTGTAGGTGCGAGCGTTAAGCTGCAACTGGAATCCGTGATGCTGGTCGAACTGGCTACCTTTGGTGGCGGTGAAGACGATTGGGCTGACGAAGTTGAAGAGAACGGCTATGTTGCCTCTTGTTCTGCCAAAGCGAGCAAACCACGCGACGAAGAAAGCTGGGACGAAGACGACGAAGAGTCCGAGGAAGCAGACGAAGACGGAGACTTCTAAGTGGAACTGTGGGAGAAAATCCTTGAGCGAATCAAGG‑T                                                                                                                                                                                                                                                                                                                  >  1:96495/1‑301`  
 `GCTCTAAGCTGAAAGTTAAATATTCTCTGGTTCCATACAAGTGGAACACTGCTGTAGGTGCGAGCGTTAAGCTGCAACTGGAATCCGTGATGCTGGTCGAACTGGCTACCTTTGGTGGCGGTGAAGACGATTGGGCTGACGAAGTTGAAGAGAACGGCTATGTTGCCTCTGGTTCTGCCAAAGCGAGCAAACCACGCGACGAAGAAAGCTGGGACGAAGACGACGCAGAGTCCGAGGAAGCAGACGAAGACGGAGACTTCTAAGTGGAACTGCGGGAGACAATCCTTGAGCGAATCAAGGg                                                                                                                                                                                                                                                                                                                   >  1:305829/1‑300`  
 `GCTCTAAGCTGAAAGTTAAATATTCTCTGGTTCCATACAAGTGGAACACTGCTGTAGGTGCGAGCGTTAAGCTGCAACTGGAATCCGTGATGCTGGTCGAACTGGCTACCTTTGGTGGCGGTGAAGACGATTGGGCTGACGAAGTTGAAGAGAACGGCTATGTTGCCTCTGGTTCTGCCAAAGCGAGCAAACCACGCGACGAAGAAAGCTGGGACGAAGACGACGAAGAGTCCGAGGAAGCAGACGACGACGGAGACTTCTAAGTGGAACTGCGGGAGAAAATCCTTGAGCGAAACAAGGg                                                                                                                                                                                                                                                                                                                   >  1:417937/1‑300`  
 `GCTCTAAGCTGAAAGTTAAATATTCTCTGGTTCCATACAAGTGGAACACTGCTGTAGGTGCGAGCGTTAAGCTGCAACTGGAATCCGTGATGCTGGTCGAACTGGCTACCTTTGGTGGCGGTGAAGACGATTGGGCTGACGAAGTTGAAGAGAACGGCTATGTTGCCTCTGGTTCTGCCAAAGCGAGCAAACCACGCGACGAAGAAAGCTGGGACGAAGACGACGAAGAGTCCGAGGAAGCAGACGAAGACGGAGACTTCTAAGTGGAACTTCGGGAGAAAATCCTTGAGCGAATCAAGG‑T                                                                                                                                                                                                                                                                                                                  >  1:347643/1‑301`  
 `GCTCTAAGCTGAAAGTTAAATATTCTCTGGTTCCATACAAGTGGAACACTGCTGTAGGTGCGAGCGTTAAGCTGCAACTGGAATCCGTGATGCTGGTCGAACTGGCTACCTTTGGTGGCGGTGAAGACGATTGGGCTGACGAAGTTGAAGAGAACGGCTATGTTGCCTCTGGTTCTGCCAAAGCGAGCAAACCACGCGACGAAGAAAGCTGGGACGAAGACGACGAAGAGTCCGAGGAAGCAGACGAAGACGGAGACTTCTAAGTGGAACTGCGGGAGAAACTCCTTGAGCGAATCAAGG‑T                                                                                                                                                                                                                                                                                                                  >  1:116669/1‑301`  
 `GCTCTAAGCTGAAAGTTAAATATTCTCTGGTTCCATACAAGTGGAACACTGCTGTAGGTGCGAGCGTTAAGCTGCAACTGGAATCCGTGATGCTGGTCGAACTGGCTACCTTTGGTGGCGGTGAAGACGATTGGGCTGACGAAGTTGAAGAGAACGGCTATGTTGCCTCTGGTTCTGCCAAAGCGAGCAAACCACGCGACGAAGAAAGCTGGGACGAAGACGACGAAGAGTCCGAGGAAGCAGACGAAGACGGAGACTTCTAAGTGGAACTGCGGGAGAAAATCCTTGAGCGAATCAAGGg                                                                                                                                                                                                                                                                                                                   >  1:621784/1‑300`  
 `GCTCTAAGCTGAAAGTTAAATATTCTCTGGTTCCATACAAGTGGAACACTGCTGTAGGTGCGAGCGTTAAGCTGCAACTGGAATCCGTGATGCTGGTCGAACTGGCTACCTTTGGTGGCGGTGAAGACGATTGGGCTGACGAAGTTGAAGAGAACGGCTATGTTGCCTCTGGTTCTGCCAAAGCGAGCAAACCACGCGACGAAGAAAGCTGGGACGAAGACGACGAAGAGTCCGAGGAAGCAGACGAAGACGGAGACTTCTAAGTGGAACTGCGGGAGAAAATCCTTGAGCGAATCAAGG‑T                                                                                                                                                                                                                                                                                                                  <  1:32351/301‑1`  
 `GCTCTAAGCTGAAAGTTAAATATTCTCTGGTTCCATACAAGTGGAACACTGCTGTAGGTGCGAGCGTTAAGCTGCAACTGGAATCCGTGATGCTGGTCGAACTGGCTACCTTTGGTGGCGGTGAAGACGATTGGGCTGACGAAGTTGAAGAGAACGGCTATGTTGCCTCTGGTTCTGCCAAAGCGAGCAAACCACGCGACGAAGAAAGCTGGGACGAAGACGACGAAGAGTCCGAGGAAGCAGACGAAGACGGAGACTTCTAAGTGGAACTGCGGGAGAAAATCCTTGAGCGAATCAAGG‑T                                                                                                                                                                                                                                                                                                                  <  1:9030/301‑1`  
 `GCTCTAAGCTGAAAGTTAAATATTCTCTGGTTCCATACAAGTGGAACACTGCTGTAGGTGCGAGCGTTAAGCTGCAACTGGAATCCGTGATGCTGGTCGAACTGGCTACCTTTGGTGGCGGTGAAGACGATTGGGCTGACGAAGTTGAAGAGAACGGCTATGTTGCCTCTGGTTCTGCCAAAGCGAGCAAACCACGCGACGAAGAAAGCTGGGACGAAGACGACGAAGAGTCCGAGGAAGCAGACGAAGACGGAGACTTCTAAGTGGAACTGCGGGAGAAAATCCTTGAGCGAATCAAGG‑T                                                                                                                                                                                                                                                                                                                  >  1:239799/1‑301`  
 `GCTCTAAGCTGAAAGTTAAATATTCTCTGGTTCCATACAAGTGGAACACTGCTGTAGGTGCGAGCGTTAAGCTGCAACTGGAATCCGTGATGCTGGTCGAACTGGCTACCTTTGGTGGCGGTGAAGACGATTGGGCTGACGAAGTTGAAGAGAACGGCTATGTTGCCTCTGGTTCTGCCAAAGCGAGCAAACCACGCGACGAAGAAAGCTGGGACGAAGACGACGAAGAGTCCGAGGAAGCAGACGAAGACGGAGACTTCTAAGTGGAACTGCGGGAGAAAATCCTTGAGCGAATCAAGG‑T                                                                                                                                                                                                                                                                                                                  >  1:610912/1‑301`  
 `GCTCTAAGCTGAAAGTTAAATATTCTCTGGTTCCATACAAGTGGAACACTGCTGTAGGTGCGAGCGTTAAGCTGCAACTGGAATCCGTGATGCTGGTCGAACTGGCTACCTTTGGTGGCGGTGAAGACGATTGGGCTGACGAAGTTGAAGAGAACGGCTATGTTGCCTCTGGTTCTGCCAAAGCGAGCAAACCACGCGACGAAGAAAGCTGGGACGAAGACGACGAAGAGTCCGAGGAAGCAGACGAAGACGGAGACTTCTAAGTGGAACTGCGGGAGAAAATCCTTGAGCGAATCAAGG‑T                                                                                                                                                                                                                                                                                                                  >  1:433649/1‑301`  
 `GCTCTAAGCTGAAAGTTAAATATTCTCTGGTTCCATACAAGTGGAACACTGCTGTAGGTGCGAGCGTTAAGCTGCAACTGGAATCCGTGATGCTGGTCGAACTGGCTACCTTTGGTGGCGGTGAAGACGATTGGGCTGACGAAGTTGAAGAGAACGGCTATGTTGCCTCTGGTTCTACCAAAGCGAGCAAACCACGCGACGAAGAAAGCTGGGACGAAGACGACGAAGAGTCCGAGGAAGCAGACGAAGACGGAGACTTCTAAGTGGAACTGCGGGAGACAATCCTTGAGCGAATCCAGG‑T                                                                                                                                                                                                                                                                                                                  >  1:71946/1‑301`  
 `GCTCTAAGCTGAAAGTTAAATATTCTCTGGTTCCATACAAGTGGAACACTGCTGTAGGTGCGAGCGTTAAGCTGCAACTGGAATCCGTGATGCTGGTCGAACTGGCTACCTTTGGTGGCGGTGAAGACGATTGGGCTGACGAAGTTGAAGAGAACGGCTATGTTGCATCTGGTTCTGCCAAAGCGAGCAAACCACGCGACGAAGAAAGCTGGGACGAAGACGACGAAGAGTCCGAGGAAGCAGACGAAGACGGAGACTTCTAAGTGGAACTGCGGGAGAAAATCCTTGAGCGAATCAAGG‑T                                                                                                                                                                                                                                                                                                                  <  1:385145/301‑1`  
 `GCTCTAAGCTGAAAGTTAAATATTCTCTGGTTCCATACAAGTGGAACACTGCTGTAGGTGCGAGCGTTAAGCTGCAACTGGAATCCGTGATGCTGGTCGAACTGGCTACCTTTGGTGGCGATGAAGACGATTGGGCTGACGAAGTTGAAGAGAACGGCTATGTTGCCTCTGGTTCTGCCAAAGCGAGCAAACCACGCGACGAAGAAAGCTGGGACGAAGACGACGAAGAGTCCGAGGAAGCAGACGAAGACGGAGACTTCTAAGTGGAACTGCGGGAGAAAATCCTTGAGCGAATCAAGGg                                                                                                                                                                                                                                                                                                                   >  1:225062/1‑300`  
 `GCTCTAAGCTGAAAGTTAAATATTCTCTGGTTCCATACAAGTGGAACACTGCTGTAGGTGCGAGCGGTAAGCCGCAAATGGAATCCGTGATGCTGGTCGAACTGGCTACCTTTGGTGGCGGTGAAGACGATTGGGCTGACGAAGTTGAAGAGAACGGCTATGTTGCCTCTGGTTCTGCCAAAGCGAGCAAACCACGCGACGAAGAAAGCTGGGACGAAGACGACGAAGAGTCCGAGGAAGCAGACGAAGACGGAGACTTCTAAGTGGAACTGCGGGAGAAAATCCTTGAGCGAATCAAGG‑T                                                                                                                                                                                                                                                                                                                  <  1:50881/301‑1`  
 `GCTCTAAGCTGAAAGTTAAATATTCTCTGGTTCCATACAAGTGGAACACTGCTGGAGGTGCGAGCGTTAAGCTGCAACTGGAATCCGTGATGCTGGTCGAACTGGCTACCTTTGGTGGCGGTGAAGACGATTGGGCTGACGAAGTTGAAGAGAACGGCTATGTTGCCTCTGGTTCTGCCAAAGCGAGCAAACCACGCGACGAAGAAAGCTGGGACGAAGACGACGAAGAGTCCGAGGAAGCAGACGAAGACGGAGACTTCTAAGTGGAACTGCGGGAGAAAATCCTTGAGCGAATCAAGG‑T                                                                                                                                                                                                                                                                                                                  <  1:413298/301‑1`  
 `GCTCTAAGCTGAAAGTTAAATATTCTCTGGTTCCATACAAGTGGAACACAGCTGTAGGTGCGAGCGTTAAGCTGCAACTGGAATCCGTGATGCTGGTCGAACTGGCTACCTTTGGTGGCGGTGAAGACGATTGGGCTGACGAAGTTGAAGAGAACGGCTATGTTGCCTCTGGTTCTGCCAAAGCGAGCAAACCACGCGACGAAGAAAGCTGGGACGAAGACGACGAAGAGTCCGAGGAAGCAGACGAAGAAGGAGACTTCTAAGTGGAACTGCGGGAGAAAATCCTTGAGCGAATCAAGG‑T                                                                                                                                                                                                                                                                                                                  >  1:535900/1‑301`  
 `GCTCTAAGCTGAAAGTTAAATATTCTCTGGTTCCAGACAAGTGGAACACTGCTGTAGGTGCGAGCGTTAAGCTGCAACTGGAATCCGTGATGCTGGTCGAACTGGCTACCTTTGGTGGCGGTGAAGACGATTGGGCTGACGAAGTTGAAGAGAACGGCTATGTTGCCTCTGGTTCTGCCAAAGCGAGCAAACCACGCGACGAAGAAAGCTGGGACGAAGACGACGAAGAGTCCGAGGAAGCAGACGAAGACGGAGACTTCTAAGTGGAACTGCGGGAGAAAATCCTTGAGCGAATCAAGG‑T                                                                                                                                                                                                                                                                                                                  <  1:555313/301‑1`  
 `GCTCTAAGCTGAAAGGTAAATACTCTTTGGTTCCATACAAGTGGAACACTGCTGTAGGTGCGAGCGTTAAGCTGCAACTGGAATCCGTGATGCTGGTCGAACTGGCTACCTTTGGTGGCGGTGAAGTCGATTGGGCTGACGAAGTTGAAGAGAACGGCTATGTTGCCTCTGGTTCTGCCAAAGCGAGCAAACCACGCGACGAATAAAGCTGGGACGAAGACGACGAAGAGTCCGAGGAAGCAGACGAAGACGGAGACTTCTAAGTGGAACTGCGGGAGAAAATCCTTGAGCGAATCAAGG‑T                                                                                                                                                                                                                                                                                                                  <  1:88776/301‑1`  
  `CTCTAAGCTGAAAGTTAAATATTCTCTGGTTCCATACAAGTGGAACACTGCTGTAGGTGCGAGCGTTAAGCTGCAACTGGAATCCGTGATGCTGGTCGAACTGGCTACCTTTGGTGGCGGTGAAGACGATTGGGCTGACGAAGTTGAAGAGAACGGCTATGTTGCCTCTGGTTCTGCCAAAGCGAGCAAACCACGCGACGAAGAAAGCTGGGACGAAGACGACGAAGAGTCCGAGGAAGCAGACGAAGACGGAGACTTCTAAGTGGAACTGCGGGAGAAAATCCTTGAGCGAATCAAGG‑T‑G                                                                                                                                                                                                                                                                                                                >  1:531223/1‑301`  
       `agagacagGTTAAATATTCTCTGGTTCCATACAAGTGGAACACTGCTGTAGGTGCGAGCGTTAAGCTGCAACTGGAATCCGTGATGCTGGTCGAACTGGCTACCTTTGGTGGCGGTGAAGACGATTGGGCTGACGAAGTTGAAGAGAACGGCTATGTTGCCTCTGGTTCTGCCAAAGCGAGCAAACCACGCGACGAAGAAAGCTGGGACGAAGACGACGAAGAGTCCGAGGAAGCAGACGAAGACGGAGACTTCTAAGTGGAACTGCGGGAGAAAATCCTTGAGCGAATCAAGG‑T‑GTCTCC                                                                                                                                                                                                                                                                                                           <  1:401775/293‑1`  
        `GCTGAAAGTTAAATATTCTCTGGTTCCATACAAGTGGAACACTGCTGTAGGTGCGAGCGTTAAGCTGCAACTGGAATCCGTGATGCTGGTCGAACTGGCTACCTTTGGTGGCGGTGAAGACGATTGTGCTGACGACGTTGAAGAGAACGGCTATGTTGCCTCTGGTTCTGCCAAAGCGAGCAAACCACGCGACGAAGAAAGCTGGGACGAAGACGACGAAGAGTCCGAGGAAGCAGACGAAGACGGCGACTTCTAAGTGGCACTGCGGGAGAACATCCTTGAGCGAATCTAGG‑Tctgtctt                                                                                                                                                                                                                                                                                                           >  1:552332/1‑294`  
             `AAGTTAAATATTCTCTGGTTCCATACAATTGGAATACTGCTGTAGGTGCGAGCGTTAAGCTGCAACTGGAATCCGTGATGCTGGTCGAACTGGCTACCTTTGGTGGCGGTGAAGCCGATTGGGCTGCCGAAGTTGAAGCGAACGGCTATGTTGCCTCTGGTTCTGCCAAAGCGAGCAACCCACGCGACGAAGAAAGCTGGGACGAAGACGACGAACAGTCCGAGGAAGAAGACGAAGACGGAGTCTTCTAAGTGGAACTGCGGGAGAAAATCCTTGAGCGAATCAAGG‑T‑GTCTCCCAAacg                                                                                                                                                                                                                                                                                                     >  1:62985/1‑298`  
               `GTTAAATATTCTCTGGTTCCATACAAGTGGAACACTGCTGTAGGTGCGAGCGTTAAGCTGCAACTGGAATCCGTGATGCTGGTCGAACTGGCTACCTTTGGTGGCGGTGAAGACGATTGGGCTGACGAAGTTGAAGAGAACGGCTATGTTGCCTCTGGTTCTGCCAAAGCGAGCAAACCACGCGACGAAGAAAGCTGTGACGAAGACGACGAAGAGTCCGAGGAAGCAGACGAAGACGGAGACTCCTAAGTGGAACTGCGGGAGAAAATCCTTGAGCAAATCAAGG‑T‑GTatacatgtgggg                                                                                                                                                                                                                                                                                                   >  1:134957/1‑289`  
               `GTTAAATATTCTCTGGTTCCATACAAGTGGAACACTGCTGTAGGTGCGAGCGTTAAGCTGCAACTGGAATCCGTGATGCTGGTCGAACTGGCTACCTTTGGTGGCGGTGAAGACGATTGGGCTGACGAAGTTGAAGAGAACGGCTATGTTGCCTCTGGTTCTGCCAAAGCGAGCAAACCACGCGACGAAGAAAGCTGGGACGAAGACGACGAAGAGTCCGAGGAAGCAGACGAAGACGGAGACTTCTAAGTGGAACTGCGGGAGAAACTCCTTGAGCGAATCAAGGCT‑GTCTCttatacac                                                                                                                                                                                                                                                                                                    >  1:658757/1‑293`  
               `GTTAAATATTCTCTGGTTCCATACAAGTGGAACACTGCTGTAGGTGCGAGCGTTAAGCTGCAACTGGAATCCGTGATGCTGGTCGAACTGGCTACCTTTGGTGGCGGTGAAGACGATTGGGCTGACGAAGTTGAAGAAAACGGCTATGTTGCCTCTGGTTCTGCCAAAGCGAGCAAACCACGCGACGAAGAAAGCTGGGACGAAGACGACGAAGAGTACGAGGAAGCAGACGAAGACGGAGACTTCTAAGTGGAACTGCGGGAGAAAATCCTTGAGCGAATCAAGG‑T‑GTCTCCCACCAGAC                                                                                                                                                                                                                                                                                                   >  1:288685/1‑301`  
                 `TAAATATTCTCTGGTTCCATACAAGTGGAACACTGCTGTAGGTGCGAGCGTTAAGCTGCAACTGGAATCCGTGATGCTGGTCGAACTGGCTACCTTTGGTGGCGGTGAAGACGATTGGGCTGACGAAGTTGAAGAGAACGGCTATGTTGCCTCTGGTTCTGCCAAAGCGAGCAAACCACGCGACGAAGAAAGCTGGGACGAAGACGACGAAGAGTCCGAGGAAGCAGACGAAGACGGAGACTTCTAAGTGGAACTGCGGGGGAAAATCCTTGAGCGAATCAAGGCT‑GTCTCttatacacat                                                                                                                                                                                                                                                                                                  >  1:229208/1‑291`  
                     `TATACTCTGGTTCCATACAAGTGGAACACTGCTGTAGGTGCGAGCGTTAAGCTGCAACTGGAATCCGTGATGCTGGTCGAACTGGCTACCTTTGGTGGTGGTGAAGACGATTGGGCTGACGAAGTTGAAGAGAACGGCTATGTTGCCTCTGGTTCTGCCAAAGCGAGCAAACCACGCGACGAAGAAAGCTGGGACGAAGACGACGAAGAGTCCGAGGAAGCAGACGAAGACGGAGACTTCTAAGTGGAACTGCGGGAGAAAATCCTTGAGCAAATCAAGG‑T‑GTCTCCCAACAGACTATTTG                                                                                                                                                                                                                                                                                             <  1:192494/301‑1`  
                      `ATTCTCTGGTTCCATACAAGTGGAACACTGCTGTAGGTGCGAGCGTTAAGCTGCAACTGGAATCCGTGATGCTGGTCGAACTGGCTACCTTTGGTGGCGGTGAAGACGATTGGGCTGACGAAGTTGAAGAGAACGGCTATGTTGCCTCTGGTTCTGCCAAAGCGAGCAAACCACGCGACGAAGAAAGCTGGGACGAAGACGACGAAGAGTCCGAGGAAGCAGACGAAGACGGAGACTCCTAAGTGGAACTGCGGGAGAAAATCCTTGAGCGAATCAAGG‑Tctgtctcttatacacatctcc                                                                                                                                                                                                                                                                                             >  1:372444/1‑280`  
                         `CTCTTGTTCCATACAAGTGGAACACTGCTGTAGGTGCGAGCGTTAAGCTGCAACTGGAATCCGTGATGCTGGTCGAACTGGCTACCTTTGGTGGCGGTGAAGACGATTGGGCTGACGAAGTTGAAGAGAACAGCTATGTTGCCTCTGGTTCTGCCAAAGCGAGCAAACCACGCGACGAAGAAAGCTGGGACGAAGACGACGAAGAGTCCGAGGAAGCAGACGAAGACGGAGACTTCTAAGTGGAACTGCGGGAGAAAATCCTTGAGCGAATCAAGG‑T‑GTCTCCCAACAGACTATTTGTGAT                                                                                                                                                                                                                                                                                         <  1:468984/301‑1`  
                         `CTCTGGTTCCATACAAGTGGAACACTGCTGTAGGTGCGAGCGTTAAGCTGCAACTGGAATCCGTGATGCTGGTCGAACTGGCTACCTTTGGTGGCGGTGAAGACGATTGGGCTGACGAAGTTGAAGAGAACGGCTATGTTGCCTCTGGTTCTGCCAAAGCGAGCAAACCACGCGACGAAGAAAGCTGGGACGAAGACGACGAAGAGTCCGAGGAAGCAGACGAAGACGGAGACTTCTAAGTGGAACTGCGGGAGAAAATCCTTGAGCGAATCAAGG‑Tctgtctcttatacacatctccgag                                                                                                                                                                                                                                                                                          >  1:578414/1‑277`  
                         `CTCTGGTTCCATACAAGTGGAACACTGCTGTAGGTGCGAGCGTTAAGCTGCAACTGGAATCAGTGATGCTGGTCGAACTGGCTACCTTTGGTGGCGGTGAAGACGATTGGGCTGACGAAGTTGAATAGAACGGCTATGTTGCCTCTGGTTCTGCCAAAGCGAGCAAACCACGCGACGAAGAAAGCTGGGACGAAGACGACGAAGAGTCCGAGGAAGCAGACGAAGACGGAGACTTCTAAGTGGAACTGCGGGAGAAAATCCTTGAGCGAATCAAGG‑T‑GTCTCCCAACAGACTATTTGTGct                                                                                                                                                                                                                                                                                         >  1:532474/1‑299`  
                             `GGTTCCATACAAGTGGAACACTGCTGTAGGTGCGAGCGTTAAGCTGCAACTGGAATCCGTGATGCTGGTCGAACTGGCTACCTTTGGTGGCGGTGAAGACGATTGGGCTGACGAAGTTGAAGAGAACGGCTATGTTGCCTCTGGTTCTGCCAAAGCGAGCAAATCACGCGACGAAGAAAGCTGGGACGAAGACGACGAAGAGTCCGAGGAAGCAGACGAAGACGGAGACTTCTAAGTGGAACTGCGGGAGAAAAACCTTGAGCGAATCAAGG‑Tctgtctcttatacacatctccgagccca                                                                                                                                                                                                                                                                                      >  1:589716/1‑273`  
                                      `CAAGTGGAACACTGCTGTAGGTGCGAGCGTTAAGCTGCAACTGGAATCCGTGATGCTGGTCGAACTGGCTACCTTTGGTGGCGGTGAAGACGATTGGGCTGACGAAGTTGAAGAGAACGGCTATGTTGCCTCTGGTTCTGCCAAAGCGAGCAAACCACGCGACGAAGAAAGCTGGGACGAAGACGACGAAGAGTCCGAGGAAGCAGACGAAGACGGAGACTTCTAAGTGGAACTGCGGGAGAAAATCCTTGAGCGAATCAAGG‑T‑GTCTCCCAACAGACTATTTGTGATATACGCAAAGGGA                                                                                                                                                                                                                                                                            >  1:112361/1‑301`  
                                                 `CTGCTGTAGGTGCGAGCGTTAAGCTGCAACTGGAATCCGTGATGCTGGTCGAACTGGCTACCTTTGGTGGCGGTGAAGACGATTGGGCTGACGAAGTTGAAGAGAACGGCTATGTTGCCTCTGGTTCTGCCAAAGCGGGCAAACCACGCGACGAAGAAAGCTGGGACGAAGACGACGAAGAGTCCGAGGAAGCAGACGAAGACGGAGACTTCTAAGTGGAACTGCGGGAGAAAATCCTTGAGCGAATCAAGG‑T‑GTCTCCCAACAGACTATTTGTGATATACGCAAAGGGAGGCGACATGGC                                                                                                                                                                                                                                                                 >  1:286859/1‑301`  
                                                  `TGCTGTAGGTGCGAGCGTTAAGCTGCAACTGGAATCCGTGATGCTGGTCGAACTGGCTACCTTTGGTGGCGGTGAAGACGATTGGGCTGACGAAGTTGAAGAGAACGGCTATGTTGCCTCTGGTTCTGCCAAAGCGAGCAAACCACGCGACGAAGAAAGCTGGGACGAAGACGACGAAGAGTCCGAGGAAGCAGACGAAGACGGAGACTTCTAAGTGGAACTGCGGGAGAAAATCCTTGAGCGAATCAAGG‑T‑GTCTCCCAACAGACTATTTGTGATATACGCAAAGGGAGGCGACATGGCA                                                                                                                                                                                                                                                                >  1:396126/1‑301`  
                                                   `GCTGTAGGTGCGAGCGTTAAGCTGCAACTGGAATCCGTGATGCTGGTCGAACTGGCTACCTTTGGTGGCGGTGAAGCCGATTGGGCTGACGAAGTTGAAGAGAACGGCTATGTTTCCTCTGGTTCTGCCAAAGCGAGCAAACCACGCGACGAAGAAAGCTGGGACGAAGACGACGAAGAGTCCGATGAAGCAGCCGAAGACGGAGACTTCTAAGTGGCACTTCGGGAGAAAATCCTTGAGCGAATCAAGG‑T‑GTCTCCCAACAGACTATTTGTGATCTAAGCAAAGGGAGGCGACATGGCAG                                                                                                                                                                                                                                                               >  1:243121/1‑301`  
                                                      `GTAGGTGCGAGCGTTAAGCTGCAACTGGAATCCGTGATGCTGGTCGAACTGGCTACCTTTGGTGGCGGTGAAGACGATTGGGCTGACGAAGTTGAAGAGAACGGCTATGTTGCCTCTGGTTCTGCCAAAGCGAGCAAACCACGCGACGAAGAAAGCTGGGACGAAGACGACGAAGAGTCCGAGGAAGCAGACGAAGACGGAGACTTCTAAGTGGAACTGCGGGAGAAAATCCTTGAGCGAATCAAGG‑T‑GTCTCCCAACAGACTATTTGTGATACACGCAAAGGGAGGCGACATGGCAGGTT                                                                                                                                                                                                                                                            >  1:42585/1‑301`  
                                                                               `gGGAATCCGTGATGCTGGTCGAACTGGCTACCTTTGGTGGCGGTGAAGACGATTGGGCTTACGAAGTTGAAGAGAACGGCTATGTTGCCTCTGGTTCTGACAAAGCGAGCAAACCACGCGACGAAGAAAGCTGGGACGAAGACGACGAAGAGTCCGAGGAAGCAGACGAAGACGGAGACTTCTAAGTGGAACTGCGGGAGAAAATCCTTGAGCGAATCAAGG‑T‑GTCTCACAACAGACTATTTGTGATATACGCAAAGGGAGGCGACATGGCAGGTTACGGCGCTAAAGGAATCCGAAAGGT                                                                                                                                                                                                                                   <  1:633465/300‑1`  
                                                                                                                         `GTGAAGACGATTGGGCTGACGAAGTTGAAGAGAACGGCTATGTTGCCTCTGGTTCTGCCAAAGCGAGCAAACCACGCGACGAAGAAAGCTGGGACGAAGACGACGAAGAGTCCGAGGAAGCAGACGAAGACGGAGACTTCTAAGTGGAACTGCGGGAGAAAATCCTTGAGCGAATCAAGG‑T‑GTTTCCCAACAGACTATTTGTGATATACGCAAAGGGAGGCGACATGGCAGGTTACGGCGCTAAAGGAATCCGAAAGGTTGGAGCGTTTCGCTCTGGCC‑‑TAGAGGACAAGGTTTCAAAGCA                                                                                                                                                                                       >  1:86683/1‑301`  
                                                                                                                                                 `TTGAAGGGAACGGCCATGTTGCCTCTGGTTCAGCCAAAGCGAGCAAACCACGCGACGAAGAAAGCTGGGACGAAGACGAAGAAGAGTCCGAGGAAGCAGACGAAGACGGAGACTTCTAAGTGGACCTGCGGGATAAAATCCTTGAGCGAATCAAGG‑T‑GTCTCCCAACAGACTATTTGTGATATACGCAAAGGGAGGCGACATGGCAGGTTACGGCGCTAAAGGAATCCTAAAGGTTGGAGCGTTTCGCTCTGGCC‑‑TAGAGGAAAAGGTTTCAAAGCAGTTGGAATCAAAAGGTATTAAATT                                                                                                                                                               <  1:651701/301‑1`  
                                                                                                                                                                    `ggccaaTGGTTGTGACAAAGCGAGCAAACCAAGCGACGAAGAAAGGGGGGAAGAAGACGAAGAAGAGTCAGAGGAAGAAGAAGAAGACGGAGACTTCGAAGTGGAACGGCGGGAGAAAATCCTTGAGCGAATCAAGG‑T‑GTCTCACAACAGACTATTTGTGATATACGCAAAGGGAGGCGACATGGCAGGTTACGGCGATAAAGGAATCCGAAAGGGTGGAGCGTTTCGCTCTGGCC‑‑GAGAGGACAAGGTTTCAAAGCAGTTGGAATCAAAAGGTATTAAATTCGAGTATGAAGAGTGGAAA                                                                                                                                            <  1:503676/295‑1`  
                                                                                                                                                                                    `AAAGCGAGCAAACCACGCGACGAAGAAAGCTGGGACGAAGACGACGAAGAGTCCGAGGAAGCAGACGAAGACGGAGACTTCTAAGTGGAACTGCGGGAGAAAATCCTTGAGCGAATCAAGG‑T‑GTCTCCCAACAGACTATTTGTGATATACGAAAAGGGAGGCGACATGGCAGGTTACGGCGCTAAAGGAATCCGAAAGGTTGGAGCGTTTCGCTCTGGCC‑‑TAGAGGACAAGGTTTCAAAGCAGTTGGAATCAAAAGGTATTAAATTCGAGTATGAAGAGTGGAAAGTGCCTTATGTAATTC                                                                                                                            <  1:340613/301‑1`  
                                                                                                                                                                                                  `ACGCGACGAAGAAAGCTGGGACGAAGACGACGAAGAGTCCGAGGAAGCAGACGAAGACGGAGACTTCTAAGTGGAGCTGCGGGAGAAAATCCTTGAGCGAATCAAGG‑G‑GTCTCCCAACAGACTATTTGTGATATACGCAAAGGGAGGCGACATGGCAGGTTACGGCGCTAAAGGAATCCGAAAGGTTGGAGCGTTTCGCTCTGGCC‑‑TAGAGGACAAGGTTTCAAAGCAGTTGGAATCAAAAGGTATTAAATTCGAGTATGAAGAGTGGAAAGTGCCTTATGTAATTCCGGCGAGCAATCAC                                                                                                              <  1:138080/301‑1`  
                                                                                                                                                                                                                  `TGGGAAGAAGACGACGAAGAGTCCGAGGAAGCAGACGAAGACGGAGACTTCTAAGTGGAACTGCGGGAGAAAATCCTTGGGCGAATCAAGG‑T‑GTCTCCCAACAGACTATTTGTGATATACGCAAAGGGAGGCGACATGGCAGGTTACGGCGCTAAAGGAATCCGAAAGGTTGGAGCGTTTCGCTCTGGCC‑‑TAGAGGACAAGGTTTCAAAGCAGTTGGAATCAAAAGGTATTAAATTCGAGTATGAAGAGTGGAAAGTGCCTTATGTAATTCCGGCGAGCAATCACACTTACACTCCAGACT                                                                                              <  1:370521/301‑1`  
                                                                                                                                                                                                                                  `AAGAGTCCGAGGAAGCAGACGAAGACGGAGACTTCTAAGTGGAACTGCGGGAGAAAATCCTTGAGCGAATCAAGG‑T‑GTCTCCCAACAGACTATTTGTGATATACGCAAAGGGAGGCGACATGGCAGGTTACGGCGCTAAAGGAATCCGAAAGGTTGGAGCGTTTCGCTCTGGCC‑‑TAGAGGACAAGGTTTCAAAGCAGTTGGAATCAAAAGGTATTAAATTCGAGTATGAAGAGTGGAAAGTGCCTTATGTAATTCCGGCGAGCAATCACACTTACACTCCAGACTTCTTACTTCCAAACGG                                                                              <  1:25013/301‑1`  
                                                                                                                                                                                                                                             `GAAGCAGACGAAGACGGAGACTTCTAAGTGGAACTGCGGGAGAAAATCCTTGAGCGAATCAAGG‑T‑GTCTCCCAACAGACTATTTGTGATATACGCAAAGGGAGGCGACATGGCAGGTTACGGCGCTAAAGGAATCCGAAAGGTTGGAGCGTTTCGCTCTGGCC‑‑TAGAGGACAAGGTTTCAAAGCAGTTGGAATCAAAAGGTATTAAATTCGAGTATGAAGAGTGGAAAGTGCCTTATGTAATTCCGGCGAGCAATCACACTTACACTCCAGACTTCTTACTTCCAAACGGTATATTCGTTG                                                                   >  1:107091/1‑301`  
                                                                                                                                                                                                                                                                                  `ggtgggctcggagatgtgtataagagacagGTCTCCCAACAGACTATTTGTGATATACGCAAAGGGAGGCGACATGGCAGGTTACGGCGCTAAAGGAATCCGAAAGGTTGGAGCGTTTCGCTCTGGCC‑‑TAGAGGACAAGGTTTCAAAGCAGTTGGAATCAAAAGGTATTAAATTCGAGTATGAAGAGTGGAAAGTGCCTTATGTAATTCCGGCGAGCAATCACACTTACACTCCAGACTTCTTACTTCCAAACGGTATATTCGTTGAGACAAAGGGTCTGTGGGAAAGCGATGATAGAAAG                                <  1:549824/271‑1`  
                                                                                                                                                                                                                                                                                      `gggctcggagatgtgtataagagacaGTCTCCCAACAGACTATTTGTGATATACGCAAAGGGAGGCGACATGGCAGGTTACGGCGCTAAAGGAATCCGAAAGGTTGGAGCGTTTCGCTCTGGCC‑‑TAGAGGACAAGGTTTCAAAGCAGTTGGAATCAAAAGGTATTAAATTCGAGTATGAAGAGTGGAAAGTGCCTTATGTAATTCCGGCGAGCAATCACACTTACACTCCAGACTTCTTACTTCCAAACGGTATATTCGTTGAGACAAAGGGTCTGTGGGAAAGCGATGATAGAAAGAAGC                            <  1:278577/275‑1`  
                                                                                                                                                                                                                                                                                           `TCCTTGAGCGAATCAAGG‑T‑GTCTCCCAACAGACTATTTGTGATATACGCAAAGGGAGGCGACATGGCAGGTTACGGCGCTAAAGGAATCCGAAAGGTTGGAGCGTTTCGCTCTGGCC‑‑TAGAGGACAAGGTTTCAAAGCAGTTGGAATCAAAAGGTATTAAATTCGAGTATGAAGAGTGGAAAGTGCCTTATGTAATTCCGGCGAGCAATCACACTTACACTCCAGACTTCTTACTTCCAAACGGTATATTCGTTGAGACAAAGGGTCTGTGGGAAAGCGATGATAGAAAGAAGCACTTATT                     >  1:559229/1‑301`  
                                                                                                                                                                                                                                                                                              `agagacagcctAAGG‑T‑GTCTCCCAACAGACTATTTGTGATATACGCAAAGGGAGGCGACATGGCAGGTTACGGCGCTAAAGGAATCCGAAAGGTTGGAGCGTTTCGCTCTGGCC‑‑TAGAGGACAAGGTTTCAAAGCAGTTGGAATCAAAAGGTATTAAATTCGAGTATGAAGAGTGGAAAGTGCCTTATGTAATTCCGGCGAGCAATCACACTTACACTCCAGACTTCTTACTTCCAAACGGTATATTCGTTGAGACAAAGGGTCTGTGGGAAAGCGATGATAGAAAGAAGCACTTATTAAT                  <  1:479285/290‑1`  
                                                                                                                                                                                                                                                                                               `agatCGAACCAAGG‑GGGTCTGCCAAGAGACTATTTGTGATATACGCAAAGGGGGGCGACATGGCAGGTTACGGAGCTAAAGGAATCCGAAAGGTTGGAGCGTTTCGCTCTGGCC‑‑TAGAGGACAAGGTTTCAAAGCAGTTGGAATCAAAAGGTATTAAATTCGAGTATAAAGAGTTGATAGTACCTTATGTCATTCCGGCGAACAATCACACTAACACTCCAGACTTCTTACTTCCAAACGGTAGATTCGTTTAGACAAAGGGTCTGTGGGAAAGCGATGATAGAAAGAAGCACTTATTAAT                  <  1:82034/297‑1`  
                                                                                                                                                                                                                                                                                                  `tgtataagagacagGTCTCCCAACAGACTATGTGTGATATACGCAAAGGGAGGCGACATGGCAGGTTACGGCGCTAAAGGAATCCGAAAGGTTGGAGCGTTTCGCTCTGGCCCAGAGAGGACAAGGTTTCAAAGCAGTTGGAATCAAAAGGTATTAAATTCGAGTATGAAGAGTGGAAAGTGCCTTATGTAATTCCGGCGAGCAATCACACTTACACTCCAGACTTCTTACTTCCAAACGGTATATTCGTTGAGACAAAGGGTCTGTGGGAAAGCGATGATAGAAAGAAGCACTTATTAAT                  <  1:126620/287‑1`  
                                                                                                                                                                                                                                                                                                     `tataagagacaGTCTCCCAACAGACTATTTGGGATATACGCAAAGGGGGGCGACATGGCAGGTTACGGAGCTAAAGGAATCCGAAAGGTTGGAGCGTTTCGCTCTGGCC‑‑TAGAGGACAAGGTTTCAAAGCAGTTGGAATCAAAAGGTATTAAATTCGAGTATGAAGAGTGGAAAGTGCCTTATGTAATTCCGGCGAGCAATCACACTTACACTCCAGACTTCTTACTTCCAAACGGTATATTCGTTGAGACAAAGGGTCTGTGGGAAAGCGATGATAGAAAGAAGCACTTATTAATTAGGG             <  1:320343/290‑1`  
                                                                                                                                                                                                                                                                                                      `ATCAAGG‑T‑GTCTCCCAACAGACTATTTGTGATATACGCAAAGGGAGGCGACATGGCAGGTTACGGCGCTAAAGGAATCCGAAAGGTTGGAGCGTTTCGCTCTGGCC‑‑TAGAGGACAAGGTTTCAAAGCAGTTGGAATCAAAAGGTATTAAATTCGAGTATGAAGAGTGGAAAGTGCCTTATGTAATTCCGGCGAGCAATCACACTTACACTCCAGACTTCTTACTTCCAAACGGTATATTCGTTGAGACAAAGGGTCTGTGGGACAGCGATGATAGAAAGAAGCCCTTATTCATTAGGGctg          >  1:271670/1‑298`  
                                                                                                                                                                                                                                                                                                        `aagagacaGTCTCCCAACAGACTATTTGGGATATACGCAAAGGGAGGCGACATGGCAGGTTACGGCGCTAAAGGAATCCGAAAGGTCGGAGCGTTTCGCTCTGGCC‑‑TAGAGGACAAGGTTTCAAAGCAGTTGGAATCAAAAGGTATTAAATTCGAGTATGAAGAGTGGAAAGTGCCTTATGTAATTCCGGCGAGCAATCACACTTACACTCCAGACTTCTTACTTCCAAACGGTATATTCGTTGAGACAAAGGGTCTGTGGGAAAGCGATGATAGAAAGAAGCACTTATTAATTAGGGAGC          <  1:557930/293‑1`  
                                                                                                                                                                                                                                                                                                           `ttgggGTCGCCCAACAGACTATTTGTGATAAAAGCAAAGGGAGGCGACATGGCAGGTTACGGCGCTAAAGGAATCCGAAAGGTTGGAGCGTTTCGCTCTGGCC‑‑TAGAGGACAAGGTTTCAAAGCAGTTGGAATCAAAAGGTATTAAATTCGAGTATGAAGAGTGGAAAGTGCCTTATGTAATTCCGGCGAGCAATCACACTTACACTCCAGACTTCTTACTTCCAAACGGTATATTCGTTGAGACAAAGGGTCTGTGGGAAAGCGATGATAGAAAGAAGCACTTATTAATTAGGGAACAGC       <  1:647964/296‑1`  
                                                                                                                                                                                                                                                                                                           `GG‑T‑GTCTCCCAACCGACGAATTGTGATATACGCAAAGGGAGGCGACATGGCAGGTTACGGCGCTAAAGGAATACGAAAGGTTGGAGCGTTTCGCTCTGGCC‑‑TAGAGGACAAGGTTTCAAAGCAGTTGGAATCAAAAGGTATTAAATTCGAGTATGAAGAGTGGAAAGTGCCTTATGTAATTCCGGCGAGCAATCACACTTACACTCCAGACTTCTTACTTCCAAACGGTATATTCGTTGAGACAAAGGGTCTGTGGGAAAGCGATGATAGAAAGAAGCACTTATTAATTAGGGAGCAGCAC     <  1:121561/301‑1`  
                                                                                                                                                                                                                                                                                                           `GG‑T‑GTCTCCCAACAGACTATTTGTGATATACGCAAAGGGAGGCGACATGGCAGGTTACGGCGCTAAAGGAATCCGAAAGGTTGGAGCGTTTCGCTCTGGCC‑‑TAGAGGACAAGGTTTCAAAGCAGTTGGAATCAAAAGGTATTAAATTCGAGTATGAAGAGTGGAAAGTGCCTTATGTAATTCCGGCGAGCTATCACACTTACACTCCAGACTTCTTACTTCAAAACGGTATATTCGTTGAGACACAGTGTCTGTGGGAAAGCGATGATAGAGAGAAGCACTTATTAATTAGGGCGCAGCAC     >  1:54602/1‑301`  
                                                                                                                                                                                                                                                                                                           `GG‑T‑GTCTCCCAACAGACTATTTGTGATATACGCAAAGGGAGGCGACATGGCAGGTTACGGCGCTAAAGGAATCCGAAAGGTTGGAGCGTTTCGCTCTGGCC‑‑TAGAGGACAAGGTTTCAAAGCAGTTGGAATCAAAAGGTATTAAATTCGAGTATGAAGAGTGGAAAGTGCCTTATGTAATTCCGGCGAGCAATCACACTTACACTCCAGACTTCTTACTTCCAAACGGTATATTCGTTGAGACAAAGGGTCTGTGGGAAAGCGATGATAGAAAGAAGCACTTATTAATTAGGGAGCAGCAC     <  1:196808/301‑1`  
                                                                                                                                                                                                                                                                                                           `GG‑T‑GTATCCCAACAGACTATGTGTGATATACGCAGAGGGAGGCGACATGGCAGGTTACGGCGCTAAAGGAATCCGAAAGGTTGGAGCGTTTCGCTCTGGCC‑‑TAGAGGACAAGGTTTCAAAGCAGTTGGAATCAAAAGGTATTAAATTCGAGTATGAAGAGTGGAAACTGCCTTATGTAATTCCAGCGAGCAATCACACTTACACTCCAGACTTCTTACTTCCAAACGGTATATTCGTTGAGACAAAGGGTCTGTGGGAAAGCGATGATAGAAAGAAGCACTTATTAATTAGGGAGCAGCAC     <  1:641201/301‑1`  
                                                                                                                                                                                                                                                                                                            `G‑T‑GTCTCCCAACAGACTCTTTGTGATATACGCAAAGGGAGGCGACATGGCAGGTTCCGGCGCTAAAGGAATACGAAAGGTTGGAGCGTTTCGCTCTGGCC‑‑AAGAGGACAAGGTTTCAAAGCAGTTGGAATCAAAAGGTATTAAATTCGAGTATGAAGAGTGGAAAGTGCCTTATGTAATTACGGCGAGCAATAACACTTACACTCCAGAATTCTTACTTCAAAACGGTATATTCGTTGAGCCAAAGGGTCTGTGGGAAAGAGTGGATAGAAAGAAGCACTTATCAATTAGtgctgtctctt    >  1:300213/1‑290`  
                                                                                                                                                                                                                                                                                                            `G‑T‑GTCTCCCAACAGACTATTTGTGATATACGCAAAGGGAGGCGACATGGCAGGTTACGGCGCTAAAGGAATCCGAAAGGTTGGCGCGTTTCGCTCTGGCC‑‑TAGAGGACAAGGTTTAAAAGCAGTTGGAATCAAAAGGTATTAAACTCGAGTATGAAGAGTGGAAAGTGCCTTATGTAATTCCGGCCAGCAATCACACTTACACTCCAGACTTCTTACTTCCAAACGGTATATTCGTTGAGACAAAGGGTCTGTGGGACAGCGATGATAGAAAGACGCACTTATTAATTAGctgtctcttat    >  1:541422/1‑290`  
                                                                                                                                                                                                                                                                                                            `G‑T‑GTCTCCCAACAGACTATTTGTGATATACGCAAAGGGAGGCGACATGGCAGGTTACGGCGCTAAAGGAATCCGAAAGGTTGGAGCGTTTCGCTCTGGCC‑‑TAGAGGACAAGGTTTCAAAGCAGTTGGAATCAAAAGGTTTTAAATTCGAGTATGAAGAGTGGAAAGTGCCTTATGTAATTCCGGAGAGCAATAACACTTACAATCCAGACTTTTTACTTCCAAACGGTATATTCTTTGAGACAAAGGGTCTGTGGGAAAGCGATGTTAGAAAGTAGCACTTATTACTTAGGGAGCAGCCCC    >  1:671238/1‑301`  
                                                                                                                                                                                                                                                                                                            `G‑T‑GTCTCCCAACAGACTATTTGTGATATACGCAAAGGGAGGCGACATGGCAGGTTACGGCGCTAAAGGAATCCGAAAGGTTGGAGCGTTTCGCTCTGGCC‑‑TAGAGGACAAGGTTTCAAAGCAGTTGGAATCAAAAGGTATTAAATTCGAGTATGAAGAGTGGAAAGTGCCTTATGTAATTCCGGCGAGCAATCACACTTACACTCCAGACTTCTTACTTCCAAACGGTATATTCGTTGAGACAAAGGGTCTGTGGGAAAGCGATGATAGAACGAAGCACTTATTAATTAGGGAGCAGCACC    >  1:120233/1‑301`  
                                                                                                                                                                                                                                                                                                            `G‑T‑GTCTCCCAACAGACTATTTGTGATATACGCAAAGGGAGGCGACATGGCAGGTTACGGCGCTAAAGGAATCCGAAAGGTTGGAGCGTTTCGCTCTGGCC‑‑TAGAGGACAAGGTTTCAAAGCAGTTGGAATCAAAAGGTATTAAATTCGAGTATGAAGAGTGGAAAGTGCCTTATGTAATTCCGGCGAGCAATCACACTTACACTCCAGACTTCTTACTTCCAAACGGTATATTCGTTGAGACAAAGGGTCTGTGGGAAAGCGATGATAGAAAGAAGCACTTCTTAATTAGctgtctcttat    >  1:666929/1‑290`  
                                                                                                                                                                                                                                                                                                            `G‑T‑GTCTCCCAACAGACTATTTGTGATATACGCAAAGGGAGGCGACATGGCAGGTTACGGCGCTAAAGGAATCCGAAAGGTTGGAGCGTTTCGCTCTGGCC‑‑TAGAGGACAAGGTTTCAAAGCAGTTGGAATCAAAAGGTATTAAATTCGAGTATGAAGAGTGGAAAGTGCCTTATGTAATTCCGGCGAGCAATCACACTTACACTCCAGACTTCTTACTTCCAAACGGTATATTCGTTGAGACAAAGGGTCTGTGGGAAAGCGATGATAGAAAGAAGCACTTATTAATTAGctgtctcttaa    >  1:330380/1‑290`  
                                                                                                                                                                                                                                                                                                            `G‑T‑GTCTCCCAACAGACTATTTGTGATATACGCAAAGGGAGGCGACATGGCAGGTTACGGCGCTAAAGGAATCCGAAAGGTTGGAGCGTTTCGCTCTGGCC‑‑TAGAGGACAAGGTTTCAAAGCAGTTGGAATCAAAAGGTATTAAATTCGAGTATGAAGAGTGGAAAGTGCCTTATGTAATTCCGGCGAGCAATCACACTTACACTCCAGACTTCTTACTTCCAAACGGTATATTCGTTGAGACAAAGGGTCTGTGGGAAAGCGATGATAGAAAGAAGCACTTATTAATTAGGGActgtctct    >  1:599455/1‑293`  
                                                                                                                                                                                                                                                                                                            `G‑T‑GTCTCCCAACAGACTATTTGTGATATACGCAAAGGGAGGCGACATGGCAGGTTACGGCGCTAAAGGAATCCGAAAGGTTGGAGCGTTTCGCTCTGGCC‑‑TAGAGGACAAGGTTTCAAAGCAGTTGGAATCAAAAGGTATTAAATTCGAGTATGAAGAGTGGAAAGTGCCTTATGTAATTCCGGCGAGCAATCACACTTACACTCCAGACTTCTTACTTCCAAACGGTATATTCGTTGAGACAAAGGGTCTGTGGGAAAGCGATGATAGAAAGAAGCACTTATTAATTAGGGAGCAGCACC    >  1:215833/1‑301`  
                                                                                                                                                                                                                                                                                                            `G‑T‑GTCTCCCAACAGACTATTTGTGATATACGCAAAGGGAGGCGACATGGCAGGTTACGGCGCTAAAGGAATCCGAAAGGTTGGAGCGTTTCGCTCTGGCC‑‑TAGAGGACAAGGTTTCAAAGCAGTTGGAATCAAAAGGTATTAAATTCGAGTATGAAGAGTGGAAAGTGCCTTATGTAATTCCGGCGAGCAATCACACTTACACTCCAGACTTCTTACTTCCAAACGGTATATTCGTTGAGACAAAGGGTCTGTGGGAAAGCGATGATAGAAAGAAGCACTTATTAATTAGGGAGCAGCACC    >  1:7420/1‑301`  
                                                                                                                                                                                                                                                                                                              `T‑GTCTCCCAACAGACTATTTGTGATATACGCAAAGGGAGGCGACATGGCAGGTTACGGCGCTAAAGGAATCCGAAAGGTTGGAGCGTTTCGCTCTGGCC‑‑TAGAGGACAAGGTTTCAAAGCAGTTGGAATCAAAAGGTATTAAATTCGAGTATGAAGAGTGGAAAGTGCCTTATGTAATTCCGGCGAGCAATCACACTTACACTCCAGACTTCTTACTTCCAAACGGTATATTCGTTGAGACAAAGGGTCTGTGGGAAAGCGATGATAGAAAGAAGCACTTATTAATTAGGGAGCAGCACCC   <  1:208372/301‑1`  
                                                                                                                                                                                                                                                                                                              `T‑GTCTCCCAACAGACTATTTGTGATATACGCAAAGGGAGGCGACATGGCAGGTTACGGCGCTAAAGGAATCCGAAAGGTTGGAGCGTTTCGCTCTGGCC‑‑TAGAGGACAAGGTTTCAAAGCAGTTGGAATCAAAAGGTATTAAATTCGAGTATGAAGAGTGGAAAGTGCCTTATGTAATTCCGGCGAGCAATCACACTTACACTCCAGACTTCTTACTTCCAAACGGTATATTCGTTGAGACAAAGGGTCTGTGGGAAAGCGATGATAGAAAGAAGCACTTATTAATTAGGGAGCAGCACCC   <  1:571861/301‑1`  
                                                                                                                                                                                                                                                                                                              `T‑GTCTCCCAACAGACTATTTGTGATATACGCAAAGGGAGGCGACATGGCAGGTTACGGCGCTAAAGGAATCCGAAAGGTTGGAGCGTTTCGCTCTGGCC‑‑TAGAGGACAAGGTTTCAAAGCAGTTGGAATCAAAAGGTATTAAATTCGAGTATGAAGAGTGGAAAGTGCCTTATGTAATTCCGGCGAGCAATCACACTTACACTCCAGACTTCTTACTTCCAAACGGTATATTCGTTGAGACAAAGGGTCTGTGGGAAAGCGATGATAGAAAGAAGCACTTATTAATTAGGGAGCAGCACCC   <  1:93889/301‑1`  
                                                                                                                                                                                                                                                                                                              `T‑GTCTCCCAACAGACTAATTGTGATATACGCAAAGGGAGGCGACATGGCAGGTTACGGCGCTAAAGGAATCCGAAAGGTTGGAGCGTTTCGCTCTGGCC‑‑TAGAGGACAAGGTTTCAAAGCAGTTGGAATCAAAAGGTATTAAATTCGAGTATGAAGAGTGGAAAGTGCCTTATGTAATTCCGGCGAGCAATCACACTTACACTCCAGACTTCTTACTTCCAAATGGTATATTCGTTGAGACAAAGGGTCTGTGGGAAAGCGATGATAGAAAGAAGCACTTATTAATTAGGGAGCAGCACCC   <  1:442289/301‑1`  
                                                                                                                                                                                                                                                                                                              `T‑GTCTCCCAACAGACGATTTGTGATAGACGCAAAGGAAGGCGACAAGGCAGGTTACGGCGCAAAAGGAATCCGAAAGGTTGGAGCGTTTCGCTCTGGCC‑‑TAGAGGACAAGGTTTCAAAGCAGTTGGAATCAAAAGGTATTAAATGCGAGTATGAAGAGTGGAAAGTGCCTTATGTAATTCCGGCGAGCAATCACACTTACACTCCAGACTTCTTACTTCCGAACGGTATATTCGTTGAGACAAAGGGTCTGTGGGAAAGCGATGATAGAAAGAAGCACTTATTAATTAGGGAGCAGCACCC   <  1:529101/301‑1`  
                                                                                                                                                                                                                                                                                                               `gGTCGCCCAACAGACTATTTGTGATAGACGCAAAGGGAGGAGACATTGCAGGTTACGTCGCTAAAGGAATCCGAAAGGTTGGAGCGTTTCGCTCTGGCC‑‑TAGAGGACAAGGTTTCAAAGCAGTTGGAATCAAAAGGTATTAAATTCGAGTATGAAGAGTGGAAAGTGCCTTATGTAATTCCGGCGAGCAATCTCACTTACACTCCAGACTTCTTACTTCCAAACGGTATATTCGTTGAGACAAAGGGTCTGTGGGAAAGCGATGATAGAAAGAAGCACTTATTAATTAGGGAGCAGCACCC   <  1:204286/300‑1`  
                                                                                                                                                                                                                                                                                                                `GTCTCCCAACAGACTATTTGTGATATACGCAAAGGGAGGCGACATGGCAGGTTACGGCGCTAAAGGAATCCGAAAGGTTGGAGCGTTTCGCTCTGGCC‑‑TAGAGGACAAGGTTTCAAAGCAGTTGGAATCAAAAGGTATTAAATTCGAGTATGAAGAGTGGAAAGTGCCTTATGTAATTCCTGCGAGCAATCACACTTACACTCCAGACTTCTTACTTCCAAACGGTATATTCGTTGAGACAAAGGGTCTGTGGGAAAGCGATGATAGAAAGAAGCACTTATTAATTAGGGAGCAGCACCCC  >  1:383254/1‑301`  
                                                                                                                                                                                                                                                                                                                `GTCTCCCAACAGACTATTTGTGATATACGCAAAGGGAGGCGACATGGCAGGTTACGGCGCTAAAGGAATCCGAAAGGTTGGAGCGTTTCGCTCTGGCC‑‑TAGAGGACAAGGTTTCAAAGCAGTTGGAATCAAAAGGTATTAAATTCGAGTATGAAGAGTGGAAAGTGCCTTATGTAATTCCGGCGAGCAATCACACTTACACTCCAGACTTCTTACTTCCAAACGGTATATTCGTTGAGACAAAGGGTCTGTGGGAAAGCGATGATAGAAAGAAGCACTTATTAATTAGGGAGCAGCACCtg  >  1:159156/1‑299`  
                                                                                                                                                                                                                                                                                                                `GTCTCCCAACAGACTATTTGTGATATACGCAAAGGGAGGCGACATGGCAGGTTACGGCGCTAAAGGAATCCGAAAGGTTGGAGCGTTTCGCTCTGGCC‑‑TAGAGGACAAGGTTTCAAAGCAGTTGGAATCAAAAGGTATTAAATTCGAGTATGAAGAGTGGAAAGTGCCTTATGTAATTCCGGCGAGCAATCACACTTACACTCCAGACTTCTTACTTCCAAACGGTATATTCGTTGAGACAAAGGGTCTGTGGGAAAGCGATGATAGAAAGAAGCACTTATTAATTAGGGAGCAGCACCCC  >  1:148946/1‑301`  
                                                                                                                                                                                                                                                                                                                `GTCTCCCAACAGACTATTTGTGATATACGCAAAGGGAGGCGACATGGCAGGTTACGGCGCTAAAGGAATCCGAAAGGTTGGAGCGTTTCGCTCTGGCC‑‑TAGAGGACAAGGTTTCAAAGCAGTTGGAATCAAAAGGTATTAAATTCGAGTATGAAGAGTGGAAAGTGCCTTATGTAATTCCGGCGAGCAATCACACTTACACTCCAGACTTCTTACTTCCAAACGGTATATTCGTTGAGACAAAGGGTCTGTGGGAAAGCGATGATAGAAAGAAGCACTTATTAATTAGGGAGCAGCACCCC  >  1:445632/1‑301`  
                                                                                                                                                                                                                                                                                                                `GTCTCCCAACAGACTATTTGTGATATACGCAAAGGGAGGCGACATGGCAGGTTACGGCGCTAAAGGAATCCGAAAGGTTGGAGCGTTTCGCTCTGGCC‑‑TAGAGGACAAGGTTTCAAAGCAGTTGGAATCAAAAGGTATTAAATTCGAGTATGAAGAGTGGAAAGTGCCTTATGTAATTCCGGCGAGCAATCACACTTACACTCCAGACTTCTTACTTCCAAACGGTATATTCGTTGAGACAAAGGGTCTGTGGGAAAGCGATGATAGAAAGAAGCACTTATTAATTAGGGAGCAGCACCCC  >  1:551416/1‑301`  
                                                                                                                                                                                                                                                                                                                `GTCTCCCAACAGACTATTTGTGATATACGCAAAGGGAGGCGACATGGCAGGTTACGGCGCTAAAGGAATCCGAAAGGTTGGAGCGTTTCGCTCTGGCC‑‑TAGAGGACAAGGTTTCAAAGCAGTTGGAATCAAAAGGTATTAAATTCGAGTATGAAGAGTGGAAAGTGCCTTATGTAATTCCGGCGAGCAATCACACTTACACTCCAGACTTCTTACTTCCAAACGGTATATTCGTTGAGACAAAGGGTCTGTGGGAAAGCGATGATAGAAAGAAGCACTTATTAATTAGGGAGCAGAACCCC  >  1:566544/1‑301`  
                                                                                                                                                                                                                                                                                                                `GTCTCCCAACAGACTATTTGTGATATACGCAAAGGGAGGCGACATGGCAGGTTACGGCGCTAAAGGAATCCGAAAGGTTGGAGCGTTTCGCTCTGGCC‑‑TAGAGGACAAGGTTTCAAAGCAGTTGGAATCAAAAGGTATTAAATTCGAGTATGAAGAGTGGAAAGTGCCTTATGTAATTCCGGCGAGCAATCACACTTACACTCCAGACTTCTTACTTCCAAACGGTATATTCGTTGAGACAAAGGGTCTGTGGGAAAGCGATGATAGAAAGAAGCACTCATTAATTAGGGctgtctcttat  >  1:303485/1‑290`  
                                                                                                                                                                                                                                                                                                                `GTCTCCCAACAGACTATTTGTGATATACGCAAAGGGAGGCGACATGGCAGGTTACGGCGCTAAAGGAATCCGAAAGGTTGGAGCGTTTCGCTCTGGCC‑‑TAGAGGACAAGGTTTCAAAGCAGTTGGAATCAAAAGGTATTAAATTCGAGTATGAAGAGTGGAAAGTGCCTTATGTAATTCCGGCGAGCAATCAAACTTACACTCCAGCCTTCTTACTTCCAAACGGTATATTCGTTGAGACAAAGGGTCTGTGGGAAAGCGATGATAGAAAGAAGCACTTATTAATTAGGGAGCAGCACCCC  >  1:259314/1‑301`  
                                                                                                                                                                                                                                                                                                                `GTCTCCCAACAGACTATTTGTGATATACGCAAAGGGAGGCGACATGGCAGGTTACGGAGCTAAAGGAATCCGAAAGGTTGGAGCGTTTCGCTCTGGCC‑‑TAGAGGACAAGGTTTCAAAGCAGTTGGAATCAAAAGGTATTAAATTCGAGTATGAAGAGTGGAAAGTGCCTTATGTAATTCCGGCGAGCAATCACACTTACACTCCAGACTTCTTACTTCCAAACGGTATATTCGTTGAGACAAAGGGTCTGTGGGCAAGCGATGATAGACAGACGCACTTATTctgtctcttatacacatct  >  1:2534/1‑282`  
                                                                                                                                                                                                                                                                                                                `GTCTCCCAACAGACTATTTGTGATATACGAAAAGGGAGGCGACATGGCAGGTTACGGCGCTAAAGGAATCCGAAAGGTTGGAGCGTTTCGCTCTGGCC‑‑TAGAGGACAAGGTTTCAAAGCAGTTGGAATCAAAAGGTATTAAATTCGAGTATGAAGAGTGGAAAGTGCCTTATGTAATTCCGGCGAGCAATCACACTTACACCCCAGACTTCTTACTTCCAAACGGTATATTCGTTGAGACAAAGGGTCTGTGGGAAAGCGATGATAGAAAGAAGCACTTATTAATTAGGGAGCAGCACCCC  >  1:153205/1‑301`  

                                                                                                                                                                                                                                                                                                                                                                                                                                                                                                                                                                                                                                 
`GGCTCTAAGCTGAAAGTTAAATATTCTCTGGTTCCATACAAGTGGAACACTGCTGTAGGTGCGAGCGTTAAGCTGCAACTGGAATCCGTGATGCTGGTCGAACTGGCTACCTTTGGTGGCGGTGAAGACGATTGGGCTGACGAAGTTGAAGAGAACGGCTATGTTGCCTCTGGTTCTGCCAAAGCGAGCAAACCACGCGACGAAGAAAGCTGGGACGAAGACGACGAAGAGTCCGAGGAAGCAGACGAAGACGGAGACTTCTAAGTGGAACTGCGGGAGAAAATCCTTGAGCGAATCAAGG‑T‑G‑‑‑‑‑‑‑‑‑‑‑‑‑‑‑‑‑‑‑‑‑‑‑‑‑‑‑‑‑‑‑‑‑‑‑‑‑‑‑‑‑‑‑‑‑‑‑‑‑‑‑‑‑‑‑‑‑‑‑‑‑‑‑‑‑‑‑‑‑‑‑‑‑‑‑‑‑‑‑‑‑‑‑‑‑‑‑‑‑‑‑‑‑‑‑‑‑‑‑‑‑‑‑‑‑‑‑‑‑‑‑‑‑‑‑‑‑‑‑‑‑‑‑‑‑‑‑‑‑‑‑‑‑‑‑‑‑‑‑‑‑‑‑‑‑‑‑‑‑‑‑‑‑‑‑‑‑‑‑‑‑‑‑‑‑‑‑‑‑‑‑‑‑‑‑‑‑‑‑‑‑‑‑‑‑‑‑‑‑‑‑‑‑‑‑‑‑‑‑‑‑‑‑‑‑‑‑‑‑‑‑‑‑‑‑‑‑‑‑‑‑‑‑‑‑‑‑‑‑‑‑‑‑‑‑‑‑‑‑‑‑‑‑‑‑‑‑‑‑‑‑‑‑‑‑‑‑‑‑‑‑‑‑‑‑‑‑‑‑‑‑‑‑‑‑‑‑‑‑‑‑‑‑‑‑‑‑‑‑‑‑‑‑‑‑‑‑‑‑‑‑‑  >  NC_001604.1/9593‑9895`  
`‑‑‑‑‑‑‑‑‑‑‑‑‑‑‑‑‑‑‑‑‑‑‑‑‑‑‑‑‑‑‑‑‑‑‑‑‑‑‑‑‑‑‑‑‑‑‑‑‑‑‑‑‑‑‑‑‑‑‑‑‑‑‑‑‑‑‑‑‑‑‑‑‑‑‑‑‑‑‑‑‑‑‑‑‑‑‑‑‑‑‑‑‑‑‑‑‑‑‑‑‑‑‑‑‑‑‑‑‑‑‑‑‑‑‑‑‑‑‑‑‑‑‑‑‑‑‑‑‑‑‑‑‑‑‑‑‑‑‑‑‑‑‑‑‑‑‑‑‑‑‑‑‑‑‑‑‑‑‑‑‑‑‑‑‑‑‑‑‑‑‑‑‑‑‑‑‑‑‑‑‑‑‑‑‑‑‑‑‑‑‑‑‑‑‑‑‑‑‑‑‑‑‑‑‑‑‑‑‑‑‑‑‑‑‑‑‑‑‑‑‑‑‑‑‑‑‑‑‑‑‑‑‑‑‑‑‑‑‑‑‑‑‑‑‑‑‑‑‑‑‑‑‑‑‑‑‑‑‑‑‑‑‑‑‑‑‑‑‑‑‑‑‑‑‑‑‑‑‑‑‑‑‑‑‑‑‑‑‑‑‑‑‑‑‑‑‑‑‑gg‑t‑gTCTCCCAACAGACTATTTGTGATATACGCAAAGGGAGGCGACATGGCAGGTTACGGCGCTAAAGGAATCCGAAAGGTTGGAGCGTTTCGCTCTGGCC‑‑TAGAGGACAAGGTTTCAAAGCAGTTGGAATCAAAAGGTATTAAATTCGAGTATGAAGAGTGGAAAGTGCCTTATGTAATTCCGGCGAGCAATCACACTTACACTCCAGACTTCTTACTTCCAAACGGTATATTCGTTGAGACAAAGGGTCTGTGGGAAAGCGATGATAGAAAGAAGCACTTATTAATTAGGGAGCAGCACCCC  >  NC_001604.1/10215‑10514`

  

Alignment Legend

Aligned base mismatch/match (shaded by quality score): `ATCG/ATCG` `< 3 ≤` `ATCG/ATCG` `< 10 ≤` `ATCG/ATCG` `< 22 ≤` `ATCG/ATCG` `< 34 ≤` `ATCG/ATCG` `< 38 ≤` `ATCG/ATCG`

Unaligned base: `atcg`    Masked matching base: `atcg`    Alignment gap: `‑`    Deleted base: `‑`

Reads not counted as support for junction

read\_name Not counted due to insufficient overlap past the breakpoint.

read\_name Not counted due to not crossing MOB target site duplication.
